# Effects of Disinfectants on Bacterium *Paenibacillus larvae* in Laboratory Conditions

**DOI:** 10.3390/insects15040268

**Published:** 2024-04-12

**Authors:** Ivana Tlak Gajger, Zlatko Tomljanović, Franco Mutinelli, Anna Granato, Josipa Vlainić

**Affiliations:** 1 Faculty of Veterinary Medicine, University of Zagreb, Heinzelova 55, 10000 Zagreb, Croatia; 2 Ministry of Agriculture, Ulica Grada Vukovara 78, 10000 Zagreb, Croatia; zlatko.tomljanovic@mps.hr; 3 Istituto Zooprofilattico Sperimentale delle Venezie, Viale dell’Università, 10, 35020 Legnaro, Italy; fmutinelli@izsvenezie.it (F.M.); agranato@izsvenezie.it (A.G.); 4 Institute Ruđer Bošković, Bijenička cesta 54, 10000 Zagreb, Croatia

**Keywords:** *Apis mellifera*, American foulbrood, *Paenibacillus larvae*, spores, disinfectants

## Abstract

**Simple Summary:**

American foulbrood is a highly infectious disease that can harm the beekeeping sector if it becomes clinically visible. It is caused by the bacterium *Paenibacillus larvae*, and its spores are resistant to various disinfectants. It is important to ensure effective final disinfection following eradication measures at apiary in order to prevent the disease from reoccurring. A study was conducted to test ten commercially available disinfectants commonly used in beekeeping, as well as those with proven efficacy in the medicinal and veterinary sectors, on different strains of *P. larvae* bacterium. Early diagnosis methods and proper control measures can help minimize the disease’s clinical signs and its incidence.

**Abstract:**

American foulbrood is an infectious disease of the honeybee brood that causes multiple types of damage to beekeeping. The causative agent of the disease is the bacterium *Paenibacillus larvae*, which forms resistant infective spores and is viable for decades. After the eradication measures have been implemented, in cases of clinically visible disease, it is necessary to conduct effective final disinfections of equipment and tools. This study aimed to determine the effect of ten commercially available and commonly used disinfectants on certified strains of *P. larvae* under laboratory conditions, as well as to compare the obtained results among individual genotypes of *P. larvae*. Selected products were tested by determining the zone of inhibition using an agar diffusion test, a suspension test for viable bacteria, a surface disinfectant test, and a sporicidal effect in the suspension test. Incidin OxyFoam S and Sekusept Aktiv are both effective against all examined genotypes of *P. larvae*. Despadac and Despadac Secure have a bactericidal effect, but their sporocidal effect is not as satisfactory as that of Genox. Genoll does not exhibit a sporicidal effect, and Ecocide S at 1%, Bee protect H forte, and Bee protect F did not exhibit a satisfactory sporocidal effect. Additionally, EM^®^ PROBIOTIC FOR BEES did not exhibit any bactericidal effect. The effective application of control measures and proper application of final disinfection can reduce the reoccurrence of visible clinical signs of disease, whereas methods of early diagnosis can significantly reduce the incidence of the disease.

## 1. Introduction

American foulbrood (AFB) is a severe infectious disease of honeybee colonies that threatens modern beekeeping [1]. The causative agent is the Gram-positive bacterium *Paenibacillus larvae* [2], which forms oval infectious spores. Remains of infected dead larvae contain billions of resilient spores, which can remain viable on combs, honeybee products, equipment, tools, and the apiary environment for decades. Vegetative forms of *P. larvae* are sensitive to scorching, drying, and disinfectants. According to Bakonyi et al., the infectious form of the bacterium presents as spores, and bent honeybee larva become susceptible to infection at the age when they actively take food [3]. Clinical recognition of the disease is possible based on the appearance of alterations in the honeybee brood. In the case of suspected AFB, cappings are wrinkled and retracted with dark spots and holes [4,5]. A lattice brood is noticeable, and the diseased larva turns into a shapeless, brown, and viscous ropy mass [6]. In the advanced stage, the rest of the dead larva fit along the lower wall of the honeycomb cell. A honeybee colony is officially declared infected if clinical examination has established changes typical of the disease and laboratory microscopic examination of the decayed larvae allows for identifying the *P. larvae* spores. Eradication measures involve stamping out and burning infected colonies and associated equipment, as well as the final disinfection of equipment and tools. Burning is the fastest, best, and most expensive way to combat AFB. Sometimes it is possible to save adult bees as an artificial swarm, housed in the new or disinfected hive on comb foundations [7]; however, relapses of clinically visible disease are possible due to poor implementation of final disinfection. 

Bednář et al., described various forms of physical and chemical disinfection of beekeeping tools and equipment [8]. Also, they state that the success of disinfection measures in the apiary depends on the correct choice of disinfectants, the spectrum of microorganisms, the recommended concentration of working solutions, the method of application, and exposure to the disinfectant. It is important that the material is disinfected in regard to its possible damage and the possible effect on the environment. Dobbelaere et al. refute the common opinion of beekeepers that the method of burning wooden parts of beekeeping equipment and accessories with flames is a sufficient disinfection measure [9]. Therefore, comprehensive disinfection of the wooden parts of the hive is possible by combining different methods of heat application, such as immersion of wooden parts in microcrystalline wax (150 °C, 10 min) or using high concentrations of disinfectants [10,11]. However, high concentrations of disinfectants are not economically and environmentally friendly, so implementing preventive zoo-hygienic measures and regularly replacing at least 25–30% of dark, old honeycombs is a very important way to mechanically remove *P. larvae* spores and other pathogens [12,13,14,15,16,17]. Sporicidal effects on *P. larvae* were determined for glutaraldehyde and sodium hypochlorite, 0.5% aqueous sodium hypochlorite solution, 1.1% caustic soda solution, and gamma radiation [18,19,20]. Kiriamburi et al. examined the biocidal effects of two commercial disinfectants, Virkon^®^ and Disinfection for beekeeping^®^ [21]. Furthermore, extracts of various plants (flavonoids, alkaloids, terpenes, essential oils) exerted successful in vitro inactivation of *P. larvae* in several studies [22,23,24,25,26].

The study aimed to determine the effect of ten commercially available and commonly used disinfectants in beekeeping and veterinary medicine in general, on certified strains of *P. larvae*, in laboratory conditions, and to compare the obtained results among different ERIC (enterobacterial repetitive intergenic consensus classification) genotypes.

## 2. Materials and Methods

### 2.1. Selection and Cultivation of Microorganisms

The used strains of *P. larvae* were from the German Collection of Microorganisms and Cell Cultures (DSMZ, Braunschweig, Germany): genotypes (DSM 7030 (ERIC I), DSM 25430a (ERIC II), LMG 16252 (ERIC III) and LMG 16247 (ERIC IV).

*P. larvae* strains were cultured on a solid nutrient medium Columbia sheep blood agar and in a liquid nutrient medium Brain−Heart Infusion (BHI). To prepare the liquid nutrient medium, 37 g of BHI medium, 3 g of yeast extract, and 1 L of H_2_O were used. The *P. larvae* were grown in the liquid nutrient medium on a shaker (New Brunswick Innova 4340 Incubator shaker, New Brunswick, NJ, USA) at a temperature of 37 °C and a speed of 200 rpm. The incubation period was 48 h.

### 2.2. Disinfectant Effect Test

Disinfectants were selected based on the recommendations of producers and beekeepers, as well as their availability on the market. The following disinfectants and food additives were used: Bee Protect products (Bee Protect H forte and Bee Protect F); (Honey Bee Pro | Agro Simpa d.o.o., Sisak, Croatia), which contains sucrose, macronutrients and organic acids; Genox and Genoll with foam (Genox Aquagen, Zagreb, Croatia), which contain hypochlorite acid, sodium chloride and hypochlorite ion; Despadac^®^ (Laboratories Calier, Barcelona, Spain) and Despadac Secure^®^ (Laboratories Calier, Barcelona, Spain) with active ingredients didecil-dimethyl ammonia chrysanthemum, glutaraldehyde in different ratios; Ecocid^®^ S (Krka d.d., Novo mesto, Slovenia) with active substances of potassium peroxymonosulfate, sodium dodecyl benzenesulfonate, and sulfamic acid; Sekusept^®^ Aktiv (Ecolab, Zagreb, Croatia) and Incidin^®^ Oxyfoam S (Ecolab, Zagreb, Croatia) with peracetic acid; and EM^®^ probiotic for bees (EMRO, Okinawa, Japan), which is mixture of microbials. Selected products were tested by (1) determining the zone of inhibition in the agar diffusion test, (2) suspension tests for viable bacteria, (3) surface disinfectant tests, and (4) sporicidal effects in the suspension tests for genotypes of *P. larvae* (ERIC I to ERIC IV).

#### 2.2.1. Agar Diffusion

An agar diffusion test was used to preliminarily determine which disinfectant meets the minimum criterion of reducing the number of viable bacteria. One bacterial colony was transferred from the solid nutrient medium plate to the BHI liquid nutrient medium optimized for the growth of *P. larvae* to obtain viable vegetative forms after 48 h of incubation with constant agitation. Then, bacterial cultures were diluted to 0.6 McFarland units determined by a nephelometer (Biosan Ltd., Riga, Latvia). The bacteria were diluted with a medium in a ratio of 1:9 and used in further experiments. In solid blood agar, two or more (depending on the expected diameter of the inhibition zone) wells were punctured, and individually tested disinfectants were added to them, i.e., phosphate–buffered saline (PBS), which in this case served as a negative control. To facilitate the diffusion of disinfectants into the agar, the plates were left for four hours at a temperature of 4 °C and subsequently moved to the incubator at a temperature of 37 °C. After incubation for 48 h, the diameters of the inhibition zones were measured. In further research, only substances (i.e., disinfectants) that showed efficiency in the preliminary agar diffusion test were used.

#### 2.2.2. Sporocidal Effect of Disinfectants 

Strains of *P. larvae* (ERIC I to IV) grown on solid blood agar at 30 °C were, according to the morphological peculiarities of typical colonies, suspended in 1 mL of PBS. Aliquots (0.1 mL) of bacteria were disposed of on solid blood agar plates and incubated at 30 °C for seven days to stimulate sporulation. The newly created colonies were collected with a sterile stick, suspended in PBS, and rinsed twice by centrifugation at 7500× *g* for 10 min. The resulting pellet was resuspended in PBS and kept at 4 °C until further use. The number of spores in the resulting suspension was determined spectrophotometrically (DEN-1, Biosan Ltd., Riga, Latvia) after heat treatment at 80 °C for 10 min (Thermomixer comfort, Eppendorf, Germany) to eliminate vegetative forms of bacteria. The number of spores was validated after plating of suspension and colony numbering. To determine the sporocidal effect, disinfectant was added at the concentration recommended in the manufacturer’s instructions. Exposure times were set as 5, 15, 30, and 60 mins, except for Incidin Oxy Foam S, where a 1 min exposure was added (based on manufacturer declaration). After exposing the *P. larvae* spores to disinfectant, the spore suspension was filtered (Shott’s bottle and Millipore vacuum pump used), and a filter (pore size of 0.45 μm) was placed in 2 mL of sterile PBS and vortexed (V-1, Biosan Ltd., Riga, Latvia) for two minutes to release spores. Then, 100 μL of suspension was seeded on Columbia sheep blood agar at 37 °C. After 48 h, colonies of *P. larvae* were counted, with Koch’s method of counting bacterial colonies being used to determine the number of *P. larvae* spores that survived the disinfectant exposure, which serves to determine the number of living cells on the principle that one colony grown on a solid nutrient medium indicates one live spore. Seeding using the method of dilution of the sample enabled more accurate determination and counting of the actual number of colonies grown, as the number of colonies grown corresponds to the number of bacterial cells, i.e., spores in the sample (the number of colonies is indicated as the number of colony-forming units (*Colony Forming Units*, CFUs). We mathematically obtained the exact number of viable spores as the ratio of the number of bacterial colonies divided by the volume of the planted sample and divided by the reciprocal value of the dilution planted on the substrate. The ratio of the number of bacterial colonies grown in the control and treated group creates the logarithm (Log) reduction.

#### 2.2.3. Determination of ATP Level

*P. larvae* were grown in the liquid nutrient medium BHI on the shaker to ensure ideal conditions for the growth and reproduction of the bacterial population. After the cultivation step for 48 h at 37 °C with agitation, the suspension of bacteria, prepared at a concentration of 0.6 McFarland units, was used in further research in dilution 1:10.

To determine the amount of ATP, we used the EnSURE (EnSURE Multi-Parameter Luminometer, Hygiena, Germany) instrument along with the Super Snap High Sensitivity ATP test (EnSURE, Hygiena, Germany), which detects low concentrations of ATP in the tested sample. The sampling aimed to examine the number of bacteria in the control sample before and after exposure of *P. larvae* to a disinfectant in the time sequence, depending on the time of exposure. The procedure is very simple and fast and involves immersing the test stick in the sample. According to the manufacturer’s instructions, the sample is combined with the test solution, and then placed in a luminometer where the ATP level is spectrophotometrically determined.

#### 2.2.4. Effects of the Disinfectant on the Contaminant Surface 

To determine whether the selected disinfectant works on the surface, a clean dry hard surface free from organic pollution and microorganisms was experimentally contaminated with *P. larvae*. After the surface was dried, it was treated with the selected disinfectant according to the manufacturer’s instructions regarding the concentration and length of exposure. At the end of the required time, the stick of the Super Snap High Sensitivity ATP test (Hygiena, Potsdam, Germany) was used to swab a surface of 10 cm^2^. The level of ATP was determined using an EnSURE device (Hygiena, Potsdam, Germany).

In addition, the surface was sampled with a sterile test stick (10 cm^2^), then placed in PBS and vortexed, while 100 μL of the suspension was seeded on solid blood agar and incubated for 72 h at 37 °C. After the end of incubation time, counting of bacterial colonies was used to determine the effect of a disinfectant on *P. larvae* on solid surfaces.

#### 2.2.5. Statistical Data Processing

Results were presented as the mean ± standard error of at least three repetitions of each sample and used test. As the results in the control and treated groups for all four *P. larvae* genotypes (ERIC I–IV) were the same, they are presented as one for each test performed, e.g., results for sporocidal effect for all four genotypes are presented in one column. Statistical analysis was conducted using GraphPad Prism 9.0 (GraphPad Software, La Jolla, CA, USA). The existence of a statistically significant difference between the results was determined using a one-way analysis of variance (one-way ANOVA) with Tukey’s post hoc test. 

## 3. Results

A summary of the results is presented in Table 1. All results were compared with the control groups.

**Figure 1:**
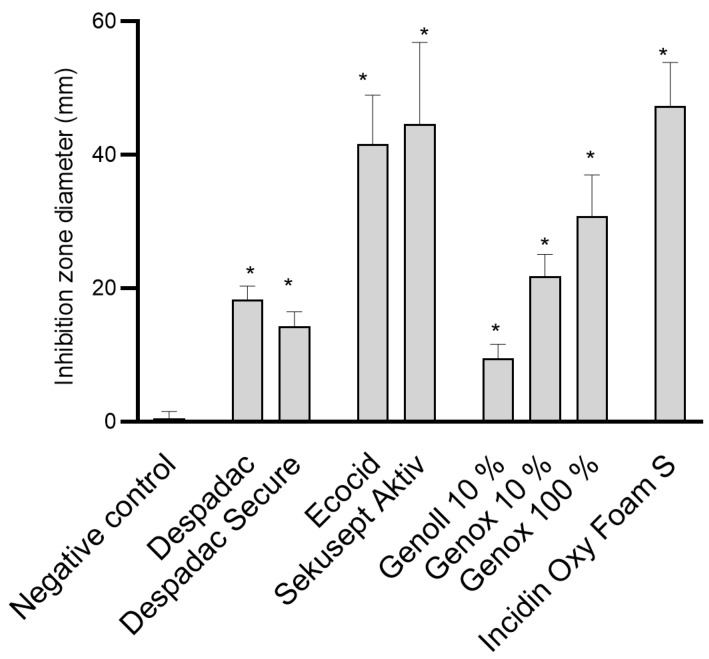
Summary of the diameter of the inhibition zone caused by examined disinfectant products on the growth of *P. larvae* bacteria. The results are shown as the mean ± standard error of at least three repetitions for each genotype. All data from the control group and for specific disinfectants are presented in one column, as the values for all genotypes (ERIC I–ERIC IV) were similar. For the sake of clarity, BeeProtect products and EM are not presented as they are not effective; * *p* < 0.0001.

Despadac (1% solution) shows inhibitory activity for the growth of *P. larvae*, and the mean diameter of the inhibition zone was 19.25 mm, while Despadac Secure (10% solution) showed an inhibition zone of 14.75 mm. These results for Despadac Secure are statistically significant (ANOVA, F (8.32) = 68.30; *p* < 0.0001) in relation to the control group. The results show that disinfectants whose main active component is active oxygen (Ecocide S, Krka, and Sekusept Aktiv, Ecolab) have a statistically significant effect on the vegetative forms, creating a relatively wide band for an inhibition zone of bacterial growth inhibition (ANOVA, F (2.17) = 35.15; *p* < 0.0001). Ecocide S caused a mean inhibition zone diameter of 41.63 ± 7.289 mm, while Sekusept Aktiv caused the formation of an inhibition zone with a width of 44.63 ± 12.19 mm. Incidin OxyFoam S caused the formation of a relatively wide band of the *P. larvae* growth inhibition zone, which was 47.52 ± 12.19 mm (*p* < 0.0001). Genox, at a concentration of 10%, showed a weaker disinfecting effect than an undiluted product; however, the producer recommends dilution of a product, even at a larger scale (up to 1% which in our study shows no efficacy,). The diameter of the inhibition zones caused by Genoll is significantly smaller (9.50 ± 1.04 mm). A single analysis of variance showed that Genox 100% and Genox 10% had a significant inhibitory effect on the growth of *P. larvae* (*p* < 0.0001) as well as Genoll (*p* = 0.0088). The application of Bee Protect F caused the formation of an inhibition zone of 24.75 ± 3.09 mm, and the application of the product Bee Protect H Forte inhibition zone of 36.75 ± 1.89 mm. Both described results compared to negative control values are statistically significant (*p* < 0.0001). The influence of effective microorganisms on the *P. larvae* showed that the visible zones of inhibition are only a few millimeters wide, so no additional analyses were made for the product EM^®^ PROBIOTIC FOR BEES. These results are presented in Figure 1.

**Figure 2:**
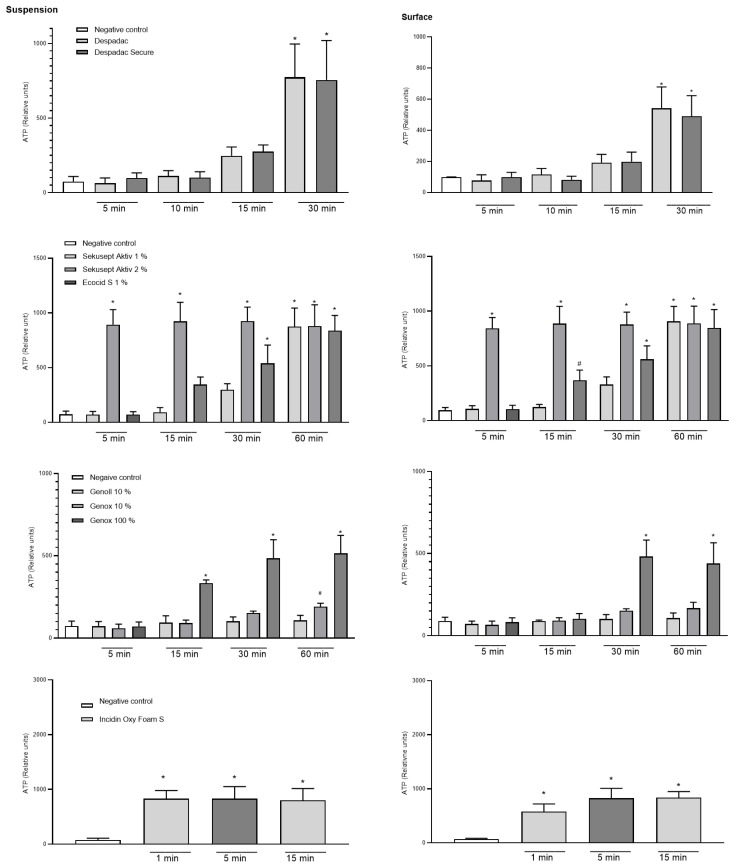
Summary of the relative units of ATP caused by disinfectant products Despadac, Sekusept Aktiv, Ecocid S, Genox and Genoll, and Incidin Oxy Foam S on the growth of *P. larvae* bacteria. Results for all genotypes treated with disinfectant are merged into one column. The results are shown as the mean ± standard error of at least three repetitions; * *p* < 0.0001; # *p* < 0.001.

In the suspension test with the vegetative form of *P. larvae*, the effectiveness of the disinfectant during exposure following the standards for bactericidal action was determined. The results show that both Despadac products show statistically significant bactericidal actions on *P. larvae* (indirectly measured by the amount of ATP in suspension) (ANOVA, F (8.31) = 26.74; *p* < 0.0001). More precisely, after 15 min of exposure of the bacteria to Despadac disinfectant, the value of the relative units of ATP increased to 247.50 ± 29.55, reaching 775.50 ± 110.70 after 30 min of exposure. After exposure to Despadac Secure for 15 min, an increase in relative ATP units to 275.50 ± 22.16 was observed, reaching 755.0 ± 132.60 after 30 min. The results show that active oxygen-based products have a statistically significant bactericidal effect on *P. larvae* (ANOVA, F (12.44) = 46.18, *p* < 0.0001) depending on the time of exposure. In Sekusept Aktiv assets at 2% concentration, the effect was significant after 5 min of exposure (893.80 ± 68.66 RU, *p* < 0.0001) and in 1% concentration after 30 min (297.50 RU ± 28.10; *p* < 0.0001). The action of Ecocid S was observed after 15 min (346.50 ± 33.98) and after 30 min (539.80 ± 83.54) (*p* < 0.0001). After 60 min of exposure to vegetative forms of *P. larvae* at both applied concentrations of Sekusept Aktiv, as well as Ecocide S, a very similar number of relative units was determined, which indicates their significant bactericidal action (values of relative units as follows: 875.50 ± 84.95, 880.0 ± 97.21 and 838.50 ± 69.65; *p* < 0.0001). A statistically significant effect was caused by the disinfectant Sekusept Aktiv in 2% concentration after 5 min of exposure (*p* < 0.0001), while Sekusept Aktiv in 1% concentration and Ecocid S showed a statistically significant effect after 30 min (*p* < 0.0001). For Incidin OxyFoam S, the measured value after 1 min (exposure time referred by manufacturer) was 830.70 ± 61.39 (*p* < 0.0001), then 830.70 ± 83.70 after 5 min, while, after 15 min, a significant increase was observed to 801.10 ± 81.21 (ANOVA F (3.20) = 18.05, *p* < 0.0001). Genoll did not affect *P. larvae,* and the values of the relative units in the time frame of treatment were as follows: 72.33 ± 12.82; 93.20 ± 19.02; 101.60 ± 12.25 and 107.50 ± 15.47, respectively. Similarly, Genox’s 10% was efficient at the longest period of exposure (191.30 ± 10.33, *p* = 0.03). On the other hand, undiluted Genox significantly affected bacteria after 15 min (333.50 ± 10.24; *p* < 0.0001), 30 min (483.50 ± 56.76; *p* < 0.0001) and after 60 min of exposure (513.50 ± 54.94; *p* < 0.0001). The results of the action of Bee Protect H Forte on the vegetative form of *P. larvae* showed that there was a significant increase in the amount of free ATP, which is an indicator of the decomposition of *P. larvae*. Consequently, there was a statistically significant decrease in the number of bacteria (ANOVA, F (2.15) = 13.79; *p* = 0.0004). Results are presented in Figure 2. 

A statistically significant difference between the experimental and control group was determined after surface treatment for 30 min with Despadac (543.0 ± 67.77) and Despadac Secure (490.00 ± 66.71) (ANOVA, *p* < 0.0001; for both groups) for all genotypes of *P. larvae.* A significant decrease in the number of *P. larvae* after surface treatment with Ecocid S was observed after 15 min (340.00 ± 32.90; *p* = 0.0006), and after 30 and 60 min of exposure, respectively, an even more significant increase in the number of relative ATP units was observed (542.80 ± 82.94; 840.20 ± 68.55). Sekusept Aktiv disinfectant (1%) significantly reduces the number of viable *P. larvae* after 60 min (875.50 ± 84.95; *p* < 0.0001). After one minute of surface treatment with Incidin OxyFoam S disinfectant, the number of *P. larvae* was significantly reduced (577.70 ± 57.72, *p* < 0.0001), and a further decrease was observed after 5 min (826.60 ± 68.11, *p* < 0.0001) and 15 min (836.40 ± 42.58, *p* < 0.0001), respectively. A significant effect on reducing the number of bacteria was observed by Genox only in 100% concentration after 30 min (481.80 ± 40.86; *p* < 0.0001) and 60 min of action (440.40 ± 46.95, *p* < 0.0001). After treating the surface with the Bee Protect line, even after 60 min of exposure, there was no increase in the value of the relative number of ATP units, nor a decrease in the number of viable bacteria. Results are presented in Figure 2.

The sporicidal effect of disinfectants from the Despadac group was not observed after 10 min exposure, while, after 30 min, it amounted to a one Log reduction for Despadac Secure, i.e., a two Log reduction for Despadac. After 60 min of action of both disinfectants, the number of spores was reduced by 99%, i.e., disinfectants caused a reduction in the number of spores by two logarithms. Disinfectants with oxygen as an active substance show a sporicidal effect after 30 min, namely a six Log reduction for Sekusept Aktiv (2%) and three Log reduction for Ecocid S (1%). After 60 min of action, both Ecocid S and Sekusept Aktiv saw a six and five Log reduction, respectively, while Sekusept Aktiv at 1% concentration reduces the number of spores only by one logarithm. After application of Incidin Oxy Foam S, an active substance hydrogen peroxide, the sporocidal effect after 30 min of exposure was six logarithms. The results showed that the disinfectant Genoll has no sporicidal effect. Genox at 10% caused a reduction in the number of spores of *P. larvae* by one logarithm, as well as at 100% concentration for three logarithms during exposure lasting 30 and 60 min, respectively. Bee Protect products do not show a sporicidal or negligible effect, as they also reduce the number of spores by less than one logarithm during exposure of 60 min. All four genotypes of *P. larvae* (ERIC I to ERIC IV) in the sporicidal suspension tests showed equal sensitivity to all the biocides tested.

**Figure 3:**
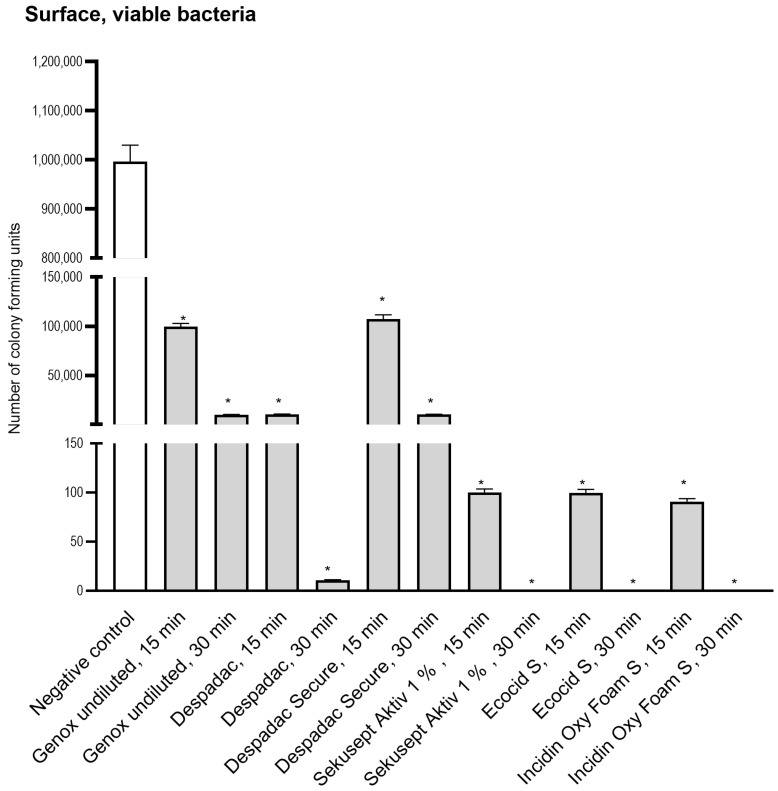
Results of a number of Colony Forming Units following application of disinfectant onto the surface. For the sake of clarity, results obtained following 5 and 60 min are omitted as they are comparable to those obtained after 15 and 30 min, which are presented. All genotypes produced were susceptible to disinfectants at the same level and thus are grouped into one column. The statistically significant decrease for CFU for all groups was * *p* < 0.0001 versus control group.

We applied 1 million microorganisms onto a dry and smooth surface (square 10 cm^2^) and, following disinfectant action, found a reduced number of viable bacteria (using CFU method) for all disinfectants applied (Figure 3). We found the reduction to 100672 CFU for undiluted Genox (100%) after 15 min and 9976 CFU after 30 min, which was similar to the reduction achieved after application of Despadac Secure. The decreases in Despadac reached 10025 CFU and 11 CFU, respectively. Sekusept Aktiv (1%) and Ecocid s (1%) reduced the number of viable bacteria after 15 min for 4 Log (cca 100 CFU remained viable) and after 30 min for 6 Log (cca 1 CFU remained). Incidin OxyFoam S at decreased the number of CFU: after 15 min to 90.70 and after 30 min to 1, corresponding to a five and six Log reduction.

## 4. Discussion

Although AFB is a notifiable disease present all over the world, till now there have been no uniform measures nor guidelines that address the effectiveness of disinfectants against bacteria *P. larvae*; thus, we performed a series of experiments using several products with different active ingredients to find the most appropriate for use in apiaries, keeping in mind effectiveness, user-friendly features, and the overall cost.

Hypochlorous acid is an active ingredient in sodium hypochlorite that reacts quickly to various substances, such as proteins, DNA, lipids, thiols, and disulfides. It is believed that this reaction prevents the germination of *P. larvae* spores by affecting the inner membrane of exposed spores [26].

This study found that two disinfectant products, Genox and Genoll, had an inhibitory effect on vegetative forms of *P. larvae* in an agar diffusion test. The result for Genox depended on the concentration applied. In the suspension test, undiluted Genox had a significant inhibitory effect after 15 min of administration that was dependent on the duration of exposure. A similar result was observed on surfaces after 30 min that was not dependent on contact time. It is important to note that the effect of Genox weakened slightly after 60 min, as all chlorine-based biocides have a time-limited effect due to wear during exposure to environmental factors. Hypochlorite-based disinfectants are also consumed more in the presence of organic matter, and their effectiveness is affected by the presence of proteins, nucleic acids, or other organic substances in the environment [27].

The microbicidal effect of chlorine is achieved by damaging functional molecules, such as DNA or proteins [28], and depends on the oxidative potential. A study found that dry spores of bacteria are more sensitive to NaOCl than wet spores [18] and that there are no germinating spores after 40 min of exposure in dry spores. In contrast, in a humid environment, an effective concentration of only above 0.025% of active chlorine was found. However, in a study of sporicidal action on *P. larvae* microenvironments in wet conditions, Genox 10% reduces the number of germinating spores by one logarithm, or Genox 100% for three logarithms after 30 min of contact. This disinfectant does not have a desirable sporicidal profile due to the long time it takes to achieve a sporicidal effect, which is also very limited. To achieve a good sporicidal activity, it would be expected to reduce the number of spores by a minimum of five Log reduction in real contact time. Sporicidal activity of 30 min or more requires immersion of contaminated equipment in a disinfectant solution, making disinfection difficult, time consuming, and relatively expensive.

The antimicrobial properties of hydrogen peroxide are attributed to the creation of hydroxyl radicals. When present in sufficient concentrations, hydroxyl radicals can harm nucleic acids, proteins, and lipids [29]. Killing spores with hydrogen peroxide does not involve damaging their DNA, unlike vegetative forms of bacteria. In a study of commercially available disinfectants with different chemical compositions, we selected the product Incidin OxyFoam S, as it has a declared sporicidal action and a stabilized hydrogen peroxide content of 1.5 g per 100 g of liquid. The results showed that this disinfectant product, following the manufacturer’s declaration, has antimicrobial effects on vegetative forms of *P. larvae* after just one minute of exposure in both suspension and surface tests. Additionally, the sporicidal action of Incidin OxyFoam S was determined to reduce by six Log after 30 min of contact. 

A previously published study revealed that a 7% solution of hydrogen peroxide can inactivate spores of bacteria after six hours of exposure [30]. In another study, authors found that spores of *Bacillus* spp. exposed to 10% hydrogen peroxide for one hour were unable to germinate [31]. However, these studies were conducted using hydrogen peroxide that was not mixed with other substances or auxiliary agents, which is different from our research. No formulations of hydrogen peroxide that are stabilized and contain additional agents for oxidation and enhanced action have been patented and proven to be more effective. They act on the proteins from spore sheaths, create hydroxyl free radicals, and oxidize membrane lipids, enabling the disinfectant to act inside the cell, affecting vegetative forms of bacteria.

Hydrogen peroxide is highly effective in killing bacteria and spores in different environments, such as hospitals and industrial settings. It is used in the form of gas through dispersing and nebulization devices, either alone or in combination with silver. Although the mechanism of inactivation of bacteria and spores by hydrogen peroxide gas is complex and not fully understood, research has shown that the gas penetrates deeper into spore structures, leading to the oxidation of essential amino acids required for spore germination [32].

In the domestic market, only one product has been declared safe for bees and used in beekeeping. This study focuses on two available products—Despadac and Despadac Secure—both of which contain didecyl-dimethyl ammonium chloride (as quaternary ammonium salts of the 3rd generation; 1955) and glutaraldehyde as active components. Quaternary ammonium salts have been in use for many years in hand antiseptics, disinfectants, and preservatives in wood processing or for the preparation of eye drops. They have a wide range of possible chemical structures that can effectively reduce the number of bacteria or inhibit and suppress their reproduction. The findings of this study indicate that the Despadac product line exhibited bactericidal properties after the bacteria were exposed for 30 min to this disinfectant.

However, there was only a slight decrease in the number of germinating spores of *P. larvae*, ranging from one or two logarithms. Such a result is not entirely satisfactory for disinfection purposes, but it may suggest that Despadac products could be used for sanitation. Similarly, a previous study showed that 0.06% of third-generation quartile ammonium salt in the Carrier test did not have a disinfectant effect on the spores *Bacillus stearothermophilus* [33]. This suggests that dodecyl-dimethyl may not be effective against spores, as the spore sheath is composed of proteins, such as keratin and biguanides, on which quartile ammonium chloride does not act [18]. Quaternary ammonium salts are used as biocides, and they work by interacting with the cytoplasmic membrane of bacteria. This interaction results in a range of processes, such as adsorption on the cell wall, penetration into the cell, reaction with the cytoplasmic membrane (lipid or protein), and disorganization of the membrane. These processes lead to leakage of intracellular material, degradation of proteins and nucleic acids, and ultimately, cell wall lysis caused by autolytic enzymes [34]. It has been shown that the selection of formulations and methods of application of disinfection affects the effectiveness of quaternary ammonium salts, and relatively few studies have been conducted in which their effectiveness is evaluated in practical conditions. In addition, we should consider the possible determination of chemical residues (ecological dynamics) and the emergence of resistance to repeatedly applied disinfectants [35]. However, the research objectives did not meaningfully confirm the possible occurrence of resistance to the action of quaternary ammonium salts because they do not have fully comparable data, as quaternary ammonium salts exist today in seven generations and, in combination with other substances (commercial preparations), can have a changed effect.

According to Regulation (EC) No 396/2005 of the European Parliament and the Council [36] (ANON, 2005), didecyldimethylammonium-chloride has been authorized as an active substance in plant protection products for use only on ornamental plants. However, its authorization has been revoked, as its use may lead to the appearance of residues in food for humans and animals. As it has been observed that their use in plant protection products leads to the appearance of residues in food, minimum residue levels have been proposed. Considering that disinfectant products used in beekeeping must be safe because honeybee colonies produce food that must not contain residues of a chemical origin, we believe that Despadac products are not a suitable choice for disinfection in beekeeping. Moreover, we have not found any record of these products being registered as biocides in the Republic of Croatia, raising questions about their regular distribution that require further investigation. In addition, this active substance is not listed in the regulation (EU) No 528/2012 of the European Parliament and the Council [37] concerning making biocidal products available on the market.

The study indicates that disinfectants containing peracetic acid are the most effective against vegetative forms and spores of *P. larvae* bacteria. These disinfectants have been tested in independent microbiological laboratories and have shown bactericidal properties compared to rod bacteria, as well as sporicidal action in relatively short exposures. Peracetic acid is a potent biocide even at low concentrations ranging from 0.0001% to 0.2%.

Peracetic acid shows an advantage over other types of active substance disinfectants because it remains effective even in the presence of organic residues and is broken down into non-toxic and non-mutagenic substances—acetic acid and oxygen. Thus, it ensures a high degree of disinfection effect in a short contact period. We used these desirable properties of disinfectants, along with the stability of the prepared solution over a long period, to investigate the effect of two commercial products on the vegetative forms of *P. larvae* and their spores. Notably, researchers found that the action of peracetic acid as a disinfectant is not dependent on the temperature of the environment, although, at higher temperatures, the results are manifested by weaker antimicrobial activity [38]. The results from the same study showed that, during the entire experimental period of 24 days, Sekusept Aktiv solution had the same disinfection potential for at least four consecutive days. Studies have also shown that the effectiveness of peracetic acid is different depending on whether the microorganisms are in suspension or on the surface, which was not the case in our research. Kunigk et al., (2001) determined that the kinetics of bacterial cell destruction are twofold and may correspond to the contact time of peracetic acid from 2 to 25 min, and the other 25 to 35 min of contact [39]. Similar to ours is a study in which the effectiveness of peracetic acid was shown, which was constant in the first 30 min of contact of disinfectant with bacteria, but subsequently increased [40].

Sekusept Aktiv is a powder used in human medicine to disinfect instruments; however, in veterinary medicine, it is not yet widely used. Unlike this product, and the same active component, Ecocid S, produced by the Slovenian company Krka, has been present on the market for many years, and as such it is relatively often used as a disinfectant in veterinary medicine. Research shows that bactericidal concentrations of Ecocid S disinfectant cause the destruction of the cell wall and cytoplasmic membrane of mycobacteria, as synonymous with a microorganism with very high resistance to disinfectants and external factors. The results also showed that Ecocid S causes decay of the granular components of the cytoplasm with the formation of fine granular inclusions and vacuoles. These irreversible changes in the structural elements of mycobacteria led to the deterioration of bacterial cells [41]. 

The study of Kiriamburi et al., aimed to examine and compare the biocidal effect of two disinfectant products: “Disinfection for beekeeping” (DFB) (Swienty, Sønderborg, Denmark) and Virkon S (Lanxess, Berlin, Germany) on *P. larvae* spores [22]. Previous studies have shown that the 1% solution of Virkon (active ingredient potassium peroxymonosulfate) has a bactericidal effect on *Pseudomonas aeruginosa*, *Escherichia coli*, *Staphylococcus aureus*, *Enterococcus hirae* and *Mycobacterium smegmatis* in suspension tests and Carrier tests on *P. aeruginosa*, *E. coli*, *S. aureus* and *E. hirae*. However, this same concentration of Virkon did not show an inhibitory effect on spores and fungi, i.e., research has shown that the concentration and required time are not following the guidelines for sporicidal and fungicidal action [42]. In their paper, the same authors concluded that 1% Virkon is effective only against vegetative bacteria, yeasts, and viruses, and should therefore be considered a low-level disinfectant [42]. The improved formulation of Virkon S has been shown in studies to kill up to 80% of *P. larvae* spores [6]; however, in the Kiriamburi et al., study, the biocidal effect ranged from 88.6 to 96.8% after 30 min of treatment duration [22]. 

The suspension test showed that Bee protect H forte had a bactericidal effect, but the test on surfaces did not show the same effect. Additionally, the Bee protect products did not meet the set standards for an overall sporicidal effect in the suspension test. Although these products can increase the effect with the time of exposure of bacteria to disinfectant, they are not recommended for use in the final disinfection of equipment, accessories, and apiary after the sanitation of clinically visible AFB. However, they can be used as an aid in disinfection despite being declared as food supplements for bees. 

It should be emphasized that, in disinfecting terms, reducing the number of spores of *P. larvae* by two logarithms (99%) does not represent a sufficient degree of disinfection due to the presence of a large number of spores. In addition, by reducing their number, it may be possible to reduce the extent of infection and/or the rate of development of the disease within one honeybee colony, but it certainly does not lead to a significant reduction in the incidence of the disease.

Effective microorganisms (EMRO, Kishaba, Japan) contain dozens of strains of microorganisms (bacteria, fungi, and mold) that are considered both beneficial for the physiological functioning of the organism and achieving balance in the environment and nature. Effective microorganisms are used in agriculture, forestry, animal husbandry, aquaculture, beekeeping, environmental protection and medicine [43,44,45,46]. The benefit of microorganisms in water purification have been documented for many years [43], as well as in the reduction in unpleasant odors on farms [47]. It has also been shown that this formulation of microorganisms applied in tumor cell culture leads to cell apoptosis [48]. In this study, the effect was investigated by using a dietary supplement for bees EM^®^ PROBIOTIC FOR BEES. The achieved inhibitory effect was minimal, and therefore this product cannot be considered to be a disinfectant or biocide. However, the action of effective microorganisms in a living organism is multifaceted, as this product works to preserve normal microflora in the gastrointestinal system of mammals and insects based on the exclusion of pathogenic microorganisms and competitive antagonistic action [49] by changing metabolic pathways through enhancing the action of digestive enzymes while reducing the activity of bacterial enzymes and ammonia formation [50] and having positive effects on the immune system and gut microbiome of bees [51].

## 5. Conclusions

The products Incidin OxyFoam S and Sekusept Aktiv (when used at a concentration of 2%) have demonstrated a satisfactory sporicidal effect on all four genotypes (ERIC I to ERIC IV) of *P. larvae*. However, Despadac and Despadac Secure showed a bactericidal effect, but their sporicidal effect is not as satisfactory as that of Genox. On the other hand, the product Genoll with foam does not exhibit any sporicidal effect; additionally, the products Ecocide S at a concentration of 1%, as well as Bee protect H forte and Bee protect F, did not exhibit a satisfactory sporicidal effect on *P. larvae*. The food additive EM^®^ PROBIOTIC FOR BEES did not exhibit a bactericidal effect. Therefore, we strongly recommend considering the effectiveness of a disinfectant before use and choosing an appropriate option to reduce the reoccurrence of a disease in apiary.

## Figures and Tables

**Table 1:** Summary of results on disinfectant effects of ten commercially available products on bacterium *P. larvae* for all examined genotypes (ERIC I–ERIC IV).

Bacterium *Paenibacillus larvae*	Method for Determination of Disinfectant Effects										
Disinfectant product	Inhibition zone diameter (mm)	Suspension test for viable bacteria				Surface disinfectant test				Sporicidal suspension test	
		Determination of the amount of ATP (ATP units)								Logarithms of reducing spores (log 10)	
		5	15	30	60	5	15	30	60	30	60
		Exposure to disinfectant (min)									
Bee Protect H forte	36.75	-	-	-	172.00	-	-	-	-	-	<1
Bee Protect F	24.75	-	-	-	102.20	-	-	-	-	-	<1
Genox 100%	30.10	71.80	333.50	483.50	513.50	76.00	102.10	481.80	440.40	3	3
Genox 10%	23.20	64.20	90.40	151.20	175.60	61.10	120.40	154.30	170.20	1	1
Genoll 10%	9.50	72.33	93.20	101.60	107.50	68.00	94.20	103.40	120.30	-	-
Despadac	19.25	52.40	247.50	775.50	-	84.20	182.20	543.00	-	2	-
Despadac Secure	14.75	98.80	275.50	755.00	-	102.60	192.40	490.00	-	1	-
Ecocid S	41.63	80.20	346.50	539.80	838.50	50.80	340.00	542.80	840.20		6
Sekusept aktiv 2%	44.63	893.80	942.40	930.00	875.50	843.20	896.80	860.00	864.20	6	5
Sekusept aktiv 1%	-	75.20	102.00	297.50	880.00	52.40	68.20	360.40	875.60	3	1
Incidin Oxyfoam S	47.52	830.70	801.10	-	-	826.60	836.40	-	-	6	6
EM^®^ probiotic for bees	-	-				-				-	

## Data Availability

Data were presented in the manuscript.
